# Parental Reflective Functioning as a Moderator of the Link Between Prematurity and Parental Stress

**DOI:** 10.3389/fpsyt.2022.804694

**Published:** 2022-02-23

**Authors:** Daphna G. Dollberg, Yael Harlev, Sivan Malishkevitch, Yael Leitner

**Affiliations:** ^1^School of Behavioral Sciences, The Academic College of Tel Aviv-Yaffo, Tel Aviv, Israel; ^2^Child Development Center, Dana-Dwek Children's Hospital, Sourasky Medical Center, Tel Aviv, Israel

**Keywords:** parental reflective functioning, parent mentalization, prematurity, parenting stress, PDI interview

## Abstract

We examined group differences between parents, both mothers and fathers, of premature and full-term infants to determine whether they differed in their reports of subjective parenting stress and in their level of parental reflective functioning (PRF). We also tested whether each parent's reflective functioning moderated the links between birth status (prematurity vs. full-term) and parenting stress. A sample of 73 cohabiting, heterosexual Israeli families with a premature (28–36th week gestational age, *N* = 34) or full-term infant (37th week and above gestational age, *N* = 39) participated, comprising the two parents' groups. Infants' age averaged 7.07 months (SD = 1.28). Each parent completed the Parent Stress Inventory (PSI) individually to determine his/her subjective personal and childrearing stress levels. The Parent Development Interview (PDI-R2-S) was used to obtain each parent's PRF (self and child/relation-focused) level. Findings showed that the premature and full-term parents did not differ in their PSI scores or PRF levels. However, mothers' self-focused PRF moderated the link between prematurity and personal parenting stress, whereas fathers' self-focused PRF moderated the link between prematurity and childrearing parenting stress. Furthermore, fathers' and mothers' PRF operated differently in the premature and full-term parents' groups. The findings highlight the importance of mothers' and fathers' PRF in predicting parents' subjective stress in general and particularly in the case of infant prematurity. We discuss these findings and their relevance for preventive and therapeutic perinatal interventions.

## Introduction

Current developmental thinking stresses the transactional and intertwined influences between the infant and the parents in shaping the infant's developmental outcome (1) and the parents' interactive patterns and parenting experiences (2). In line with this transactional framework, parenting stress is understood as reflecting the interplay between the infant's characteristics such as prematurity, and the parents' resources such as their parental reflective functioning (PRF). We examine how prematurity and PRF contribute to and interact with each other in predicting parenting stress.

According to the World Health Organization (3), an infant is considered premature if born before completing 37 weeks of gestation. Preterm birth is considered a risk factor for the infant, potentially negatively affecting several areas of the infant's development, with the risk increasing as a function of the severity of prematurity (4). Indeed, premature infants often display a variety of short- and long-term motor, language and communication, cognitive and behavioral developmental delays (5–7).

Prematurity may also put parents and their parenting experiences at risk. The premature birth usually comes unexpectedly, interfering with the normal pre- and postnatal process of parental bonding and increasing the risk of parental distress and post-traumatic symptoms (8, 9). A recent meta-analysis reported an increased risk of postpartum depression among mothers of premature infants (10). In addition, prematurity may interfere, either directly or indirectly, with the mother's parental self-image (4), parenting satisfaction and parental sense of efficacy (11).

Premature infants often display less alertness and responsiveness (12), greater passivity (13) and more temperamental difficulties (14) than full-term infants. The relative neurodevelopmental immaturity of the premature infant may make parenting more challenging than parenting a full-term infant, however, the findings in this area are inconsistent. Some studies found that parents of premature infants, particularly mothers, display less sensitive mothering (15) and engage in more controlling (16) and intrusive behaviors (17) than parents of full-term infants. Nevertheless, a recent meta-analysis reported that mothers of preterm infants were as sensitive and responsive toward their children as mothers of full term infants (18). Even less is known about fathers' parenting and prematurity. The limited evidence points to more passivity and unresponsiveness in fathers compared to mothers of premature infants (13). Fathers of premature infants also showed less synchrony during early parent-infant interactions than fathers of full-term infants (19).

Perceived parenting stress is the result of a mismatch between a parent's perceptions about available psychological and family resources and the demands of parenthood (20–22). Given the medical and developmental challenges accompanying the parenting of premature infants, we would expect greater parenting stress among these parents compared to parents of full-term infants. However, here too, the findings are inconclusive. Some studies linked prematurity with more parenting stress compared to the parenting of full-term infants (23), especially over time (24). Similarly, fathers of premature infants admitted to the intensive care unit (NICU) reported more stress than fathers of full-term infants (25). A recent meta-analysis concluded that parents of preterm-born children indeed experienced more parenting stress than parents of full-term children, however, the difference was negligible with small effect sizes (26). Moreover, mothers reported slightly more stress than fathers, but here too, the effect sizes were small (26).

One possible explanation for the lack of group differences may be related to within-group variations and mediating factors that might override between-group differences among parents of premature and full-term infants. Along these lines, mothers of premature infants often report post-traumatic distress symptoms related to the delivery and the NICU hospitalization (27) that mediate the link between premature delivery and parenting stress (28). The tendency to develop PTSD symptoms following a preterm birth may stem from medical factors related to the infant and the birth but may also depend on the personality characteristics of the parent (29). A parent's reflective functioning may be an example of such an individual parental characteristic, which may be linked to the way a parent deals with and experiences a premature birth and its related stress.

Parental reflective functioning (PRF), a particular form of the more global construct of parental mentalization, is reflected in the parent's meta-cognitive ability to view the child as a mental agent and actively observe and infer the mental states that govern and explain the child's and the parent's behaviors (30–32). PRF develops in the context of close relationships (32), is context-specific, and affects the developing parent-child relationship (30). PRF enables the parent to hold the infant's mental states in his/her own mind and to adjust the parenting to meet not only the physiological needs but also the mental needs of the infant (33). The mentalizing activity in the parent's mind is echoed by the activation of specialized neural circuits in the parental brain, specifically related to the neuropeptide Oxytocin. Doing so promotes affiliative, self-rewarding, caregiving behaviors and hence supports the infant's brain and socioemotional development (34).

Longitudinal and cross-sectional studies have linked parental mentalization in general and PRF in particular with parenting and with infant development in high and low stress conditions (35). Higher levels of parental mentalization and PRF predict favorable child developmental outcomes such as secure attachment (35), more advanced mental abilities (36), better regulation skills and fewer behavior problems (37) in children. Higher levels of PRF are associated with the mothers' ability to tolerate stress and self-regulate effectively in the face of the distress and physiological arousal created by an infant's cry (38) and with a better ability to screen out insensitive parental responses (39). On the other hand, poor PRF is associated with the mothers' insensitive parenting (40) and disruptive affective communication (41). Lower levels of prenatal PRF characterize high-risk primiparous mothers compared to those with little risk (42).

Mentalization is a multidimensional and multi-faceted capacity. Neurobiological research is increasingly converging to suggest that the capacity to mentalize is an evolutionarily prewired, species-specific human ability. The human brain is equipped with specific neuroanatomic structures and metabolic processes in which the neuropeptide Oxytocin plays a major role. In addition to being involved in caregiving and affiliative behavior, it is also involved in social cognition and mentalizing activity (43). Effective PRF involves the flexible move between automated, quick, implicit forms of reflection and more controlled, intentional modes. Those who exhibit effective PRF can combine internal and external sources of information, change their focus between the self and the other, and integrate affective and cognitive thinking (30, 33, 44). In contrast, deficient, inflexible, poor reflective functioning has been suggested as a core, transdiagnostic element in various forms of psychiatric psychopathology, including affective, thought and personality disorders (45). Moreover, PRF is context- and state-related and is therefore susceptible to the negative impact of high levels of arousal and stress (30). Heightened arousal inhibits the engagement of cortically controlled processing and interferes with the capacity for reasoning and staying engaged in the face of a child's distress. For example, mothers of infants and toddlers with early regulatory disorders reported more parental stress, which was followed by prementalizing, a form of limited, rigid, ineffective PRF (46). On the other hand, mothers with higher levels of PRF were more able to tolerate an infant's simulated cry (38). Based on these studies, researchers have suggested that PRF and stress are bidirectionally linked. Greater stress and arousal compromise one's ability to utilize PRF effectively, whereas higher levels of PRF can mitigate the negative impact of high levels of arousal (33). Indeed, evidence points to the protective nature of PRF, particularly in the context of stressful circumstances. Specifically, PRF helps mothers modulate their own arousal and contain their over-controlling behaviors (47). In one study, expecting parents' PRF mediated the link between the parents' histories of maltreatment and their ability to maintain a sense of competence as parents (48). In another study mothers' mind-mindedness (another form of parental mentalization) moderated the link between mothers' and fathers' anxiety symptoms and their children's externalizing behaviors (49). Interestingly, in this study no similar moderating effects for fathers' mind-mindedness were evident.

Most developmental studies in general, and regarding parental mentalization and PRF in particular, have focused on mothers. Consequently, little is known about fathers' PRF, whether and how it differs from the mother's PRF and how it contributes to outcomes related to parenting and the child. The existing information paints an inconsistent picture. Some evidence shows similar levels of reflective functioning regarding childhood relationships in mothers and fathers (50) and demonstrates that both parents' mentalization contributes equally to children's developmental outcomes (51, 52). Similarly, a longitudinal study that examined the impact of parents' mentalization on toddlers' behavior problems reported no gender differences in the quantity and predictive value of the fathers' and mothers' mentalization (53). In contrast, a study that looked at adolescents and their parents documented group differences between fathers' and mothers' PRF, with fathers demonstrating weaker mentalization skills than mothers (54). Cooke et al. (55) used a self-report questionnaire to assess PRF and reported that mothers' and fathers' PRF were unrelated and that mothers demonstrated more mentalization skills than fathers. The authors argued that these findings were consistent with findings regarding gender differences in theory of mind capacities (56). They also accorded with findings of normative male alexithymia, where men are less aware of emotions than women and less able to describe them (57).

Not much is known about the role of parental mentalization in general and PRF in particular in the context of prematurity. On one hand, premature infants of mothers with higher levels PRF showed the expected soothing behavior in the Still Face procedure in contrast with mothers of such infants who had poorer PRF (58), suggesting that maternal PRF is important in the context of prematurity. On the other hand, mothers of premature and full-term infants demonstrated similar frequencies of mental state talk (another form of parental mentalization) when interacting with their 3-month-old infants (4). Mothers in the two groups also reported similar levels of perceived parenting stress. Interestingly, only within the preterm group was parenting stress associated with more non-attuned mental comments, reflecting the mother's unsuccessful attempt to tease out her infant's mental state, hence, poor PRF (4).

When it comes to fathers' PRF, even less is known. Recently, Ruiz et al. (59) compared the PRF of parents of 12–20-month-old preterm and full-term infants and found no significant group, gender, or group by gender differences in the parents' PRF levels. However, the researchers reported that the fathers of premature infants showed poorer PRF than the other groups. Moreover, a qualitative analysis of the parents' responses on the PDI interview (60) revealed that mothers tended to focus more on the infants' needs and how to meet them (i.e., child-focused PRF), whereas fathers focused more on parenting activities and interests, thus demonstrating more self-focused PRF. Commenting on this study, Taubner (33) called for additional research on gender differences in PRF, particularly in the context of prematurity.

### The Current Study

It is generally agreed that parenting a premature infant, either in itself or due to the perinatal medical, psychological and developmental stressors that accompany such parenting, may be challenging for most parents. Nevertheless, studies have failed to show consistent differences in perceived parenting stress between parents of preterm and full-term infants. Furthermore, inconsistency also exists regarding the different responses of fathers and mothers to prematurity. Thus, our first goal was to examine whether parents of premature infants, both mothers and fathers, differed from parents of full-term infants in their perceived level of parenting stress. Infants born between 28 and 36 weeks' gestation were included in the preterm group. Infants born before week 28 or those with genetic abnormalities, severe neuro-functional impairment, severe neonatal complications, and neurosensory disabilities were excluded from the study because of the added unique medical complexity that may interfere with their development. Second, while PRF appears to be a relatively stable characteristic of the parent vis-a-vis a particular child, it may also be compromised by situational stress, particularly parenting stress (33). Thus, our second objective was to examine whether parents of premature infants who need to cope with the challenges that accompany prematurity exhibit lower levels of PRF compared to those in parents of full-term infants. Considering the scarce data regarding PRF and prematurity, this question was explorative in nature. We chose the specific age of roughly 6 months (corrected age in the case of premature infants) as it represents a time when developmentally, parents are learning to read and recognize their infants' internal cues (32). For parents of premature infants, this time may bring with it some relief from the immediate pressure of the unexpected delivery, the NICU experience and the uncertainty related to the premature infant's immediate medical condition, which characterizes the early months following the premature birth. On the other hand, evidence shows that less beneficial maternal interactional patterns (15) and more elevated cumulative stress (61) are already present at 6 months among mothers of preterm infants as compared to full-term infants.

Furthermore, commenting on Ruiz's study, Taubner (33) argued that whereas the PDI provides an overall measure of PRF, it is important to explore specific facets of PRF in the context of prematurity. Based on this suggestion, we examined the parent's self-focused PRF and the child and relation-focused PRF separately.

Finally, given emerging evidence regarding the protective nature of PRF and its potential mitigating effect on stress, our third goal was to examine whether mothers' and fathers' PRF moderated the association between prematurity and parents' subjective stress about parenting. Given the inconsistent findings regarding the moderating impact of fathers' and mothers' mentalization, we examined the mothers' and fathers' PRF separately, allowing us to examine their unique moderating effects.

We tested three hypotheses.

H1: Mothers and fathers of premature and full-term infants will differ in their reports of parenting stress.
H2: Mothers and fathers of premature and full-term infants will differ in their PRF.
H3: The mother's and father's PRF will each moderate the association between prematurity and parental stress so that this association will differ depending on each parent's gender and PRF.

## Methods

### Participants

The sample consisted of 73 cohabiting, heterosexual Israeli families (73 mothers and 73 fathers) with a 6–7-month-old infant (age corrected for the premature infants). Of the sample, 34 families had a premature infant (born between the 28th−36th gestation week and weighing 842–2,270 grams) and 37 families had a full-term infant (born after the 37th gestation week and weighing 2,000–4,095 grams). Thirty-eight of the infants were girls (52.05%) and the majority (74.29%) were first born. The families lived in the center of Israel, and most parents were native born (90% of mothers and 88.24% of fathers). Mothers' mean age was 32.30 years (range 24–48, SD = 3.79) and fathers' mean age was 33.78 years (range 26–53, SD = 4.24). Household income varied, with 74.3% reporting an above average income, 10% an average income and 15.7% a below average income according to Israeli standards. All of the parents were at least high school graduates, with fathers reporting an average of 16.17 years of education (SD = 2.23) and mothers reporting an average of 16.68 years of education (SD = 1.64). Most infants were cared for at home by their parents or a paid caregiver (65.71%), whereas the remainder were in home care facilities such as daycare programs. Table 1 presents the demographics and developmental information for the premature and full-term infant groups.

**Table 1:** Demographics and infants' developmental information for the premature and full-term infants' groups.

	**Premature infants group**	**Full-term infants group**	***t*** **/χ^2^**
	**(***n*** = 34)**	**(***n*** = 39)**	
	***M*** **(SD)**	***M*** **(SD)**	
Infant's chronological age (months)*	8.09 (0.98)	6.22 (0.79)	−8.86**
Birth week	31.38 (2.0)	39.18 (1.29)	19.03**
Birth weight (g)	1,479.81 (368.29)	3,158.55 (478.07)	16.22**
Mother's age	33.03 (4.23)	31.68 (3.31)	−1.49
Mother's education (years)	16.29 (1.6)	17 (1.63)	1.80
Father's age	35.47 (4.59)	32.28 (3.28)	−3.32**
Father's education (years)	16.26 (2.45)	16.09 (2.17)	−0.30
	***N*** **(%)**	***N*** **(%)**	
Mother's country of origin: Israel	28 (87.50%)	35 (92.11)	0.41
Father's country of origin : Israel	27 (84.38%)	33 (91.67)	0.87
**Household income:**			
Below average	2 (6.3%)	9 (23.68)	
Average	2 (6.3%)	5 (13.16)	
Above average	28 (87.5%)	24 (63.16)	5.58
Infant's gender: girl	17 (50%)	21 (53.8)	0.11
Infant's birth order: first	21 (65.6%)	31 (81.6)	6.53
**Caregiving arrangement:**			
Family/nanny	21 (65.63%)	25 (65.79)	0
Daycare	11 (34.38%)	13 (34.21)	

* *The chronological age of the premature infants is before age correction for prematurity*.

** *p < 0.01*.

### Materials and Procedure

This study is part of a larger cross-sectional study examining the parenting experience, family functioning and developmental outcomes of premature and full-term infants. Volunteering families were recruited from the Child Developmental Center at the Tel Aviv Sourasky Medical Center and from the community via social network posts. Interested parents were contacted by phone and following their oral agreement to participate, a home visit was scheduled. During the home visit, both parents signed a consent form and completed electronic questionnaires independently. Two research assistants conducted the home visit during which they interviewed the parents individually using the Parent Development Interview Revised-Short Form (PDI-R2-SF). The interviews were audio recorded and later transcribed and coded as detailed below.

The study was approved by the hospital's (TLV-0287-13) and Tel Aviv-Yaffo Academic College's (2014024) IRBs.

### Measures

#### Parental Reflective Functioning

Parental reflective functioning (PRF) was assessed with the Parent Development Interview-Revised-Short Form [PDI-R2-S; (60)], a 35-item semi-structured interview lasting about an hour and assessing parents' representations of their infant and the relationship with him/her. Sample items include “Describe a time in the last week when you and (child's name) really 'clicked;” “Now, describe a time in the last week when you and (child's name) really weren't 'clicking;” “Tell me about a time in the last week or two when you felt really angry as a parent...What kind of effect do these feelings have on your child?” The PDI-R-2S was translated into Hebrew by the first author, who was trained on using it by Prof. Arietta Slade, the instrument's lead developer. The Addendum to the Reflective Functioning Scoring Manual (62) was used to obtain the parents' PRF scores. The scale has 11 points ranging from −1 (negative PRF) to 9 (full or exceptional PRF), where scores below 5 represent a negative, absent or low PRF, and scores of 5 and above represent average to high levels of PRF. Following earlier research (47, 63) and to get a better and deeper understanding of the parents' PRF (33), two additional factors were calculated: the child/relation-focused PRF, which consisted of averaging eight items regarding the parent's view of the child's mental states and relational needs (e.g., “When your child is upset, what does he/she do? How does that make you feel? What do you do?”) and the self-focused PRF, which was computed by averaging seven items referring to the parent's self-reflection regarding his/her own mental state (e.g., “What gives you the most pain or difficulty in being a parent?”). Two advanced graduate psychology students coded the transcribed responses under the supervision of the first author. Pre-coding interrater reliability was computed based on 10 training interviews with an intraclass correlation coefficient (ICC) of 0.80. Interrater reliability was measured again on 10 randomly selected mothers' and fathers' interviews during the coding process with an average ICC of 0.71. An adequate internal reliability was found for the self-focused PRF (Cronbach's alpha = 0.77, 0.79 for mothers and fathers, respectively) and the child/relation-focused PRF (Cronbach's alpha = 0.60, 0.72 for mothers and fathers, respectively). The coders were blind to the mothers' scores on the other measures used in the study.

#### Parenting Stress

Parenting stress was assessed with the Parenting Stress Index-Short Form [PSI-SF, (20)]. This is a self-report questionnaire consisting of 36 items that measure the parent's subjective distress involved in parenting. It links parenting responsibilities and the parent-child relationship. Participants respond to items on a Likert scale ranging from 1 (“strongly agree”) to 5 (“strongly disagree”), reflecting the degree to which the parent agrees with the specific statement regarding personal feelings. Prior research (64) has demonstrated the utility of obtaining scores regarding two factors. The first is “personal distress”, which includes 12 items and reflects the parent's personal concerns and negative feelings about parenting (e.g., “I feel trapped by my responsibility as a parent” or “I don't enjoy things as I used to”). The second factor is “childrearing stress”, which includes 24 items concerning the stress related to raising children. It deals with the infant's regulatory capacities and the parent's expectations regarding the parent-child relationship (e.g., “I feel that my baby is moody and gets upset easily” or “I feel that my infant doesn't smile as other infants do”). Scores for the two factors are obtained by averaging the relevant items for each factor, with higher scores reflecting greater stress. We found good internal reliability for the personal distress factor (Cronbach's alpha = 0.82, 0.75 for the mothers and fathers, respectively) and for the childrearing stress factor (Cronbach's alpha = 0.94, 0.91 for the mothers and the fathers, respectively).

#### The Families' Demographics and Child Developmental History

The families' demographics and child developmental history were obtained from the demographic questionnaire that each family completed. The questionnaire asked about the parents' age, education level, country of birth and household income. Questions also referred to the infant's delivery, health and developmental history and current care setting.

### Data Analysis

We analyzed the data using IBM SPSS Statistics version 25 and the PROCESS version 3.0 macro for SPSS (65). Prior to testing the hypotheses, we examined the descriptive statistics for all of the variables to ensure their normal distribution. We used Little's Missing Completely at Random Test (MCAR) to determine what data were missing at random and replaced them using the means of the relevant variable to increase the study's power. Next, we compared the premature and full-term infant groups to examine whether they differed in their demographics. We would include significant differences between the groups as control variables when testing the model. We then computed Pearson's *r* correlations to investigate zero-order associations among the study's variables, and between them and the participants' demographics. To test H1 and H2 we used a MANOVA, comparing mothers and fathers in the premature and full-term groups on the two PSI-SF and the two PRF factors. To test H3 we used PROCESS Model 2 (65) with 5,000 bias-corrected bootstrap samples in which the mothers' and fathers' self-focused PRF and the child/relation-focused PRF moderated the links between birth status (prematurity vs. full-term) and the personal distress and childrearing stress factors. We considered the effects significant at *p* < 0.05.

## Results

The MCAR test showed a non-significant result in the PRF scores of the mothers (χ^2^ = 58.6, *p* = 0.08) and fathers (χ^2^ = 72.77, *p* = 0.52) and in the PSI-SF scores of the mothers (χ^2^ = 70.35, *p* = 0.47) and fathers (χ^2^ = 64.67, *p* = 0.66), indicating that data were missing at random. Consequently, we replaced the missing data using the means of the relevant variable according to the expectation maximization method.

We compared the premature and full-term infants' groups and found, as shown in Table 1 that the two groups did not differ significantly in their demographics except for the father's age, as fathers of preterm infants were significantly older than fathers of full-term infants. Therefore, the father's age was included in further analyses as a covariate. Expected birth-related differences were found between the premature and full-term infants so that the preterm infants had significantly lower birth weights, were born at an earlier gestational age and were older in chronological age (prior to prematurity age-correction). Next, we computed Pearson's *r* correlations among the participants' demographics and the study's variables and found no significant zero-order correlations. Finally, we computed zero-order correlations among the study's variables for the whole sample and by group. For the whole sample we found a significant positive association between a parent's personal distress and the childrearing stress PSI factors (*r* = 0.48, *p* < 0.01 for mothers and *r* = 0.22, *p* = 0.06 for fathers). We also found a strong significant positive association between a parent's self-focused PRF and the child/relation-focused PRF factors (*r* = 0.64, *p* < 0.001 for mothers and *r* = 0.72, *p* < 0.001). Moreover, there were significant associations among the spouses' PSI factors. Specifically, the mother's and father's personal distress scores were positively and moderately associated (*r* = 0.33, *p* < 0.01) as were their childrearing stress scores (*r* = 0.34, *p* < 0.01). The mothers' and fathers' self-focused PRF scores were also positively and moderately correlated (*r* = 0.28, *p* < 0.05) as were the parents' child/relation-focused PRF scores (*r* = 0.32, *p* < 0.01). Tables 2A,B present the means and standard deviations of the study's variables and the zero-order correlations between the study's variables by group (premature vs. full-term). Accordingly, in the two groups the mothers' and fathers' scores on the personal distress of the PSI correlated positively. Mothers' and fathers' scores on the PSI childrearing stress only significantly and positively correlated in the premature group but was weaker and not significant in the full-term infants' group. Concerning the association between the personal distress and childrearing stress factors within a parent, the scores correlated positively only for mothers in the two groups and were weaker insignificant in the preterm infants' group. The two stress factors did not correlate for fathers in either group. The mothers' and fathers' self-focused PRF were strongly and positively correlated in the full-term group but, interestingly, were unrelated in the preterm infants' group. The child/relation-focused PRF of the mothers and fathers correlated positively but this association did not reach significance the two groups. Finally, for both parents in the two groups there were strong positive associations between the self-focused and child/relation-focused PRF scores.

**Table 2A:** Stress level of parents of preterm infants and PRF scores: correlations and descriptive statistics (*N* = 34).

**Variables**	**1**	**2**	**3**	**4**	**5**	**6**	**7**	**8**	***M***	***Sd***
1. Mother's PD	**-**								2.43	0.74
2. Father's PD	0.34*	**-**							2.47	0.68
3. Mother's CrS	0.29	0.26	**-**						1.68	0.6
4. Father's Crs	−0.06	0.2	0.42*	**-**					1.59	0.5
5. Mother's Self PRF	−0.22	−0.26	0.09	0.23	**-**				4.18	0.96
6. Father's self PRF	−0.18	0.04	0.39*	0.43*	0.23	**-**			3.76	1.02
7. Mother-child PRF	−0.1	0.08	0.19	0.49**	0.69***	0.26	**-**		3.92	0.87
8. Father-child PRF	−0.23	0.05	0.17	0.41*	0.11	0.74***	0.33	**-**	3.80	1.11

*PD, Personal Distress Factor; CrS, Childrearing Stress Factor; Self PRF, Self-focused PRF; Child PRF, Child/relation-focused PRF*.

* *p < 0.05*.

** *p < 0.01*.

*** *p < 0.001*.

**Table 2B:** Stress level of parents of full-term infants and PRF scores: correlations and descriptive statistics (*N* = 39).

**Variables**	**1**	**2**	**3**	**4**	**5**	**6**	**7**	**8**	***M***	***Sd***
1. Mother's PD	**-**								2.61	0.71
2. Father's PD	0.34*	**-**							2.44	0.65
3. Mother's CrS	0.63***	0.29	**-**						1.84	0.67
4. Father's Crs	0.27	0.25	0.28	**-**					1.65	0.52
5. Mother's self PRF	0.3	0.05	−0.003	−0.02	**-**				4.56	0.91
6. Father's Self PRF	0.02	−0.1	−0.25	−0.23	0.46**	**-**			3.85	1.16
7. Mother-child PRF	0.1	0.12	−0.09	0.1	0.76***	0.38*	**-**		3.92	0.88
8. Father-child PRF	0.12	0.08	−0.14	−0.02	0.39*	0.79***	0.31	**-**	3.80	0.9

*PD, Personal Distress Factor; CrS, Childrearing Stress Factor; Self PRF, Self-focused PRF; Child PRF, Child/relation-focused PRF*.

* *p < 0.05*.

** *p < 0.01*.

*** *p < 0.001*.

The MANOVA showed no differences between the mothers and fathers of preterm and full-term infants on the personal distress and childrearing stress factors of the PSI (H1) and on the self-focused and child/relation-focused PRF factors (H2). Tables 2A,B lists the means and standard deviations of the variables for the mothers and fathers of the premature and full-term groups, respectively. This lack of differences was true for mothers and fathers. Hence, H1 and H2 were not supported.

To test H3 we used PROCESS Model 2 (65), in which the mothers' and fathers' self-focused PRF and the child/relation-focused PRF moderated the links between birth status (prematurity vs. full-term) and the parents' PSI stress factors. Since there was no significant difference between fathers (*M* = 2.45, SD = 0.66) and mothers [(*M* = 2.53, SD = 0.72), *t*_(72)_ = 0.81, *p* = 0.42] in their personal distress factor scores and the two were strongly correlated, and similarly, there was no significant difference in the childrearing stress factor between fathers (*M* = 1.62, SD = 0.51) and mothers [(*M* = 1.76, SD = 0.64), *t*_(72)_ = 1.83, *p* = 0.07] and the two were strongly correlated, we combined the mothers' and fathers' scores by averaging them. As a result, we obtained two joint scores: the parents' joint personal distress and the parents' joint childrearing stress. As noted above, there were also strong and significant associations between the fathers' and mothers' PRF factor scores. However, we did not combine them into a single score because we wanted to assess the moderating effect of the mothers' and fathers' PRF separately. Consequently, we tested two models predicting the parents' joint personal distress, once with the mother's and father's self-focused PRF as moderators, and a second time with the mother's and father's child/relation-focused PRF. Two additional models predicting the parents' joint childrearing stress were tested with the mother's and father's self-focused PRF and again with the mother's and father's child/relation-focused PRF. In sum, we tested four models. We also included the father's age in all of the models as a covariate.

**Figure 1:**
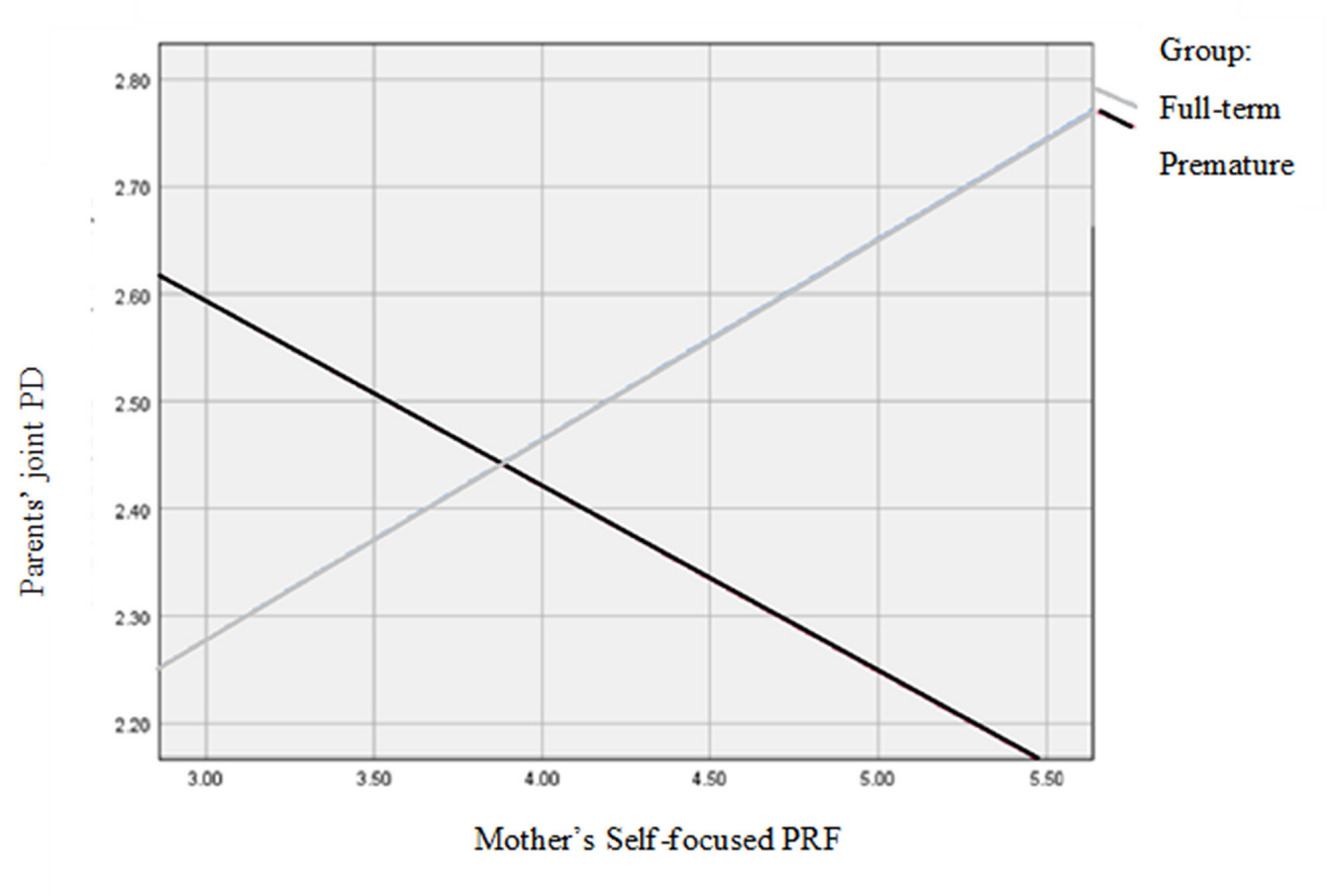
Associations between the mother's self-focused PRF and the parents' joint personal distress for the premature and full-term groups.

The model predicting personal distress from prematurity, moderated by the father's and mother's self-focused PRF, was significant [*F*_(6,61)_ = 2.21, *p* = 0.05, *R*^2^ = 0.18]. Prematurity had a direct effect on the parents' joint personal distress [*B* = 1.44, *SE* = 0.69, *t*_(68)_ = 2.08, *p* = 0.04]. Thus, parents of premature infants reported more personal distress than parents of full-term infants. The mother's self-focused PRF also had a main effect on the parents' personal distress [(*B* = 0.28, *SE* = 0.13), *t*_(68)_ = 2.21, *p* = 0.03]. However, there was also a significant interaction effect between birth status and maternal self-focused PRF [*B* = −0.45, *SE* = 0.16, *t*_(68)_ = −2.78, *p* < 0.01]. Figure 1 presents a visual illustration of the moderated links between the parents' joint personal distress and the mother's self-focused PRF for the premature and full-term groups. Accordingly, in the premature infants' group, personal distress was negatively associated with the mother's self-focused PRF. The more self-focused PRF the mother displayed, the less joint personal distress the couple reported. The reverse pattern was evident in the full-term infants' group. Here, the more self-focused PRF the mother displayed, the more joint personal distress the couple reported. The father's self-focused PRF did not add to the prediction. All of these effects were evident when controlling for the effect of the father's age [*B* = 0.04, SE = 0.02, *t*_(68)_ = 2.19, *p* = 0.03].

**Figure 2:**
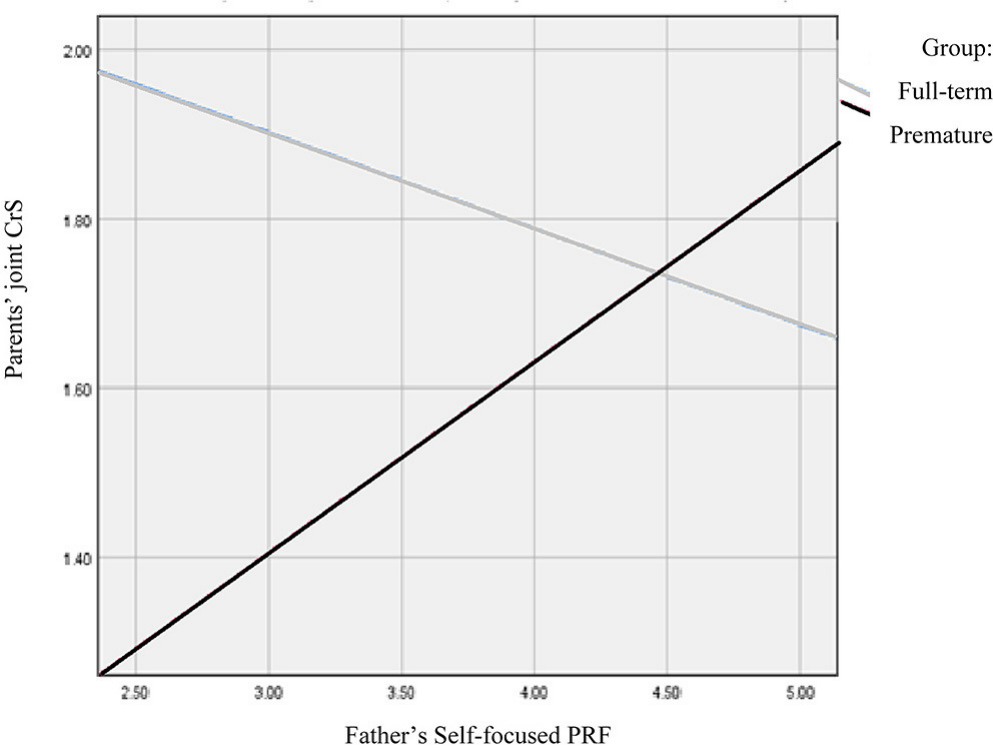
Associations between the father's self-focused PRF and the parents' joint childrearing stress for the premature and full-term groups.

The model predicting the parents' childrearing stress from prematurity moderated by the mother's and father's self-focused PRF was also significant [*F*_(6,61)_ = 3.75, *p* < 0.01, *R*^2^ = 0.27]. Prematurity had a direct effect [*B* = −1.13, *SE* = 0.58, *t*_(68)_ = −1.96, *p* = 0.05], so that parents of premature infants reported less childrearing stress than parents of full-term infants. The fathers' self-focused PRF also had a main effect on the parents' childrearing stress [*B* = −0.18, *SE* = 0.07, *t*_(68)_ = −2.41, *p* = 0.02]. However, there was also a significant interaction effect of birth status by father's PRF [*B* = 0.4, *SE* = 0.11, *t*_(68)_ = 3.71, *p* < 0.001]. Figure 2 presents a visual illustration of the moderated links between the parents' joint childrearing stress distress and the father's self-focused PRF for the premature and full-term groups. Accordingly, in the premature infants' group, childrearing stress was positively associated with the father's self-focused PRF. Thus, the more self-focused PRF the father displayed, the greater the parents' reported childrearing stress. The reverse pattern was evident in the full-term infants' group. In this group the highest levels of childrearing stress appeared in families where the father's level of self-focused PRF was lower. The mother's self-focused PRF did not add to the prediction. These effects were evident beyond the effect of the father's age [(*B* = 0.03, *SE* = 0.01), *t*_(68)_ = 2.34, *p* = 0.02].

The model predicting childrearing stress with the mother's and the father's child/relation-focused PRF as moderators was also significant [*F*_(6,61)_ = 2.3, *p* = 0.05, *R*^2^ = 0.18]. However, beyond the effect of paternal age [*B* = 0.03, *SE* = 0.02, *t*_(68)_ = 2.19, *p* = 0.03], only prematurity had a direct effect [*B* = −1.34, *SE* = 0.59, *t*_(68)_ = −2.27, *p* = 0.03]. Thus, parents of premature infants reported less childrearing stress than parents of full-term infants. There was no significant interactive effect. Finally, the model predicting parents' personal distress moderated by the mother's and the father's child/relation-focused PRF was not significant. Thus, H3 was generally supported.

## Discussion

Our first objective was to compare the levels of parenting stress and PRF among parents, both fathers and mothers, of premature and full-term infants. Contrary to our predictions, we found no group-level differences between parents of premature and full-term infants in the two factors of parenting stress we tested—childrearing stress and the parent's personal distress. Furthermore, there were differences between the parents of the preterm and full-term infants in the PRF factors we tested–the parent's self- and child/relation-focused PRF. Finally, regardless of the infant's birth status as premature or full-term, fathers and mothers did not differ in their reports of parenting stress or their PRF levels.

Next, we hypothesized that a parent's PRF would moderate the link between birth status as premature or full-term and parenting stress, such that parents with higher levels of PRF would report a weaker association between prematurity and parenting stress than parents with lower levels of PRF. We found a complex pattern of associations, partially confirming this hypothesis. Birth status and mothers' self-focused PRF predicted the parents' joint personal distress. Thus, parents of premature infants reported more joint personal distress than parents of full-term infants. In addition, higher levels of the mothers' self-focused PRF were associated with less joint personal distress in the parents. However, as hypothesized, there was an interactive effect between birth status and the mother's PRF on the parents' joint personal distress. Specifically, higher levels of the mother's self-focused PRF predicted less joint personal distress in the parents, but only in the preterm infants' group. In the full-term infants' group, the reverse pattern was observed, so that higher levels of the mother's self-focused PRF were associated with greater personal distress. No similar effect was found for the fathers' self-focused PRF. A different pattern emerged with respect to the parents' childrearing stress. Parents of premature infants reported less joint childrearing stress than parents of full-term infants. The father's self-focused PRF, but not the mother's self-focused PRF, predicted the parents' joint childrearing stress, so that higher levels of the father's self-focused PRF were linked with less childrearing stress. However, this was true only in the full-term infants' group. In the group of preterm infants, when fathers displayed higher levels of the self-focused PRF, the parents' joint childrearing stress was higher. Finally, the model predicting the parents' childrearing stress based on birth status and the parents' child/relation-focused PRF was significant. However, only birth status predicted childrearing stress, so that parents of premature infants reported less childrearing stress than parents of full-term infants. In sum, the results suggest that as predicted, a parent's PRF moderates the links between the parents' subjective reports of parenting stress. These moderated links depend on the interplay between the infant's birth status, the parent's gender and the specific PRF and stress domains tested.

The lack of group differences in parenting stress between parents of preterm and full-term infants is consistent with mounting evidence indicating no differences (4) or negligible differences (26) in parenting stress among parents of premature and full-term infants. However, they contradict other studies that did report evidence of such differences (23, 25, 66). Some researchers argue that given the advances in the quality of medical care and life-saving procedures available for premature infants, prematurity should not be viewed as stressful in and of itself (26). Rather, prematurity may become a source of stress when accompanied by exceptionally low gestational age, extremely low birth weight, traumatic delivery (10, 13, 26) and prenatal risks including high-risk pregnancies and previous losses (67). Following this argument, the lack of group differences in our study and the finding of less childrearing stress among parents in the preterm group that emerged in some of our analyses might be due to the exclusion of very sick premature infants from our sample and the high-quality pre and postnatal medical services provided in the case of prematurity in Israel [e.g., (68)]. Another factor that might have accounted for the lack of group differences in our study has to do with the infant's age. Many of the studies that did find elevated stress among parents of premature infants assessed stress shortly after the premature birth, often when the infants were still hospitalized in the NICU [e.g., (25, 69)]). Other studies have looked at parenting stress at an older age and noted an increase in parenting stress over time, possibly reflecting long-term negative effects of prematurity (24). Our decision to assess parenting stress when infants were 6–7 months old reflected our interest in understanding the parents' subjective stress when they were adjusting to their daily parenting routines and beginning to know their infant's individual psychological characteristics, meaning, developing their PRF skills vis-a-vis their infant. However, this point in time might not reflect the parents' subjective experiences at other developmental stages, when parenting a premature infant may indeed be more stressful than parenting a full-term infant. Finally, and most importantly, our findings highlighted the heterogeneity within the preterm group based on the parents' gender and PRF, which may account for the lack of between-group differences.

We found that fathers and mothers in the two groups did not differ in their subjective reports of personal and childrearing stress and their scores were mostly positively correlated. This finding contrasts with the results of Schappin et al.'s (26) meta-analysis that found slight, but significantly higher stress levels among mothers compared to fathers of premature infants. The lack of difference may reflect economic and social changes in childrearing responsibilities that have equalized mothers' and fathers' parenting stress (70).

From a psychological viewpoint, a parent's coping with parenting challenges, including prematurity, depends, at least partly, on the interplay between the parent's psychological resources and vulnerabilities (2). PRF is one such domain-specific personal characteristic. Therefore, and given the challenges accompanying the parenting of a premature infant, we hypothesized that parents of premature infants would exhibit lower levels of PRF than parents of full-term infants. Like other studies (4, 59), our hypothesis was not supported. Nevertheless, we did find that PRF moderated the links between prematurity and parenting stress. The moderating effect indicates heterogeneity and within-group variability in how parents of preterm infants cope and deal with prematurity. Specifically, we found that maternal self-focused PRF, i.e., the mother's capacity to reflect on her own mental states such as her feelings and thoughts, was a protective factor, linked with less parenting personal distress, i.e., the parent's personal concerns and negative feelings about parenting, among parents in the preterm group. This finding is consistent with mounting evidence indicating the protective and moderating role of mothers' PRF (47, 71) and maternal mentalization (49) in the face of arousal and distress. When it comes to prematurity, parents of preterm infants may be immersed in the day-to-day physical care of their premature infant, which is quite likely to be demanding and pressing. Furthermore, evidence shows that the dyadic interactions with premature infants are less rewarding than interactions with their full-term counterparts (15). In light of these challenges, it is possible that when mothers are less reflective, they focus mainly on the physical and practical needs of the premature infant which contributes to their sense of pressure and demand. They may also be preoccupied with their own worries about the infant's development and health, leading to more personal stress. In contrast, reflective mothers of premature infants may be more able to shift their attention from worrying to focusing on more benevolent aspects of their parenting (30). In doing so, they may make space for the experience of delight and pride in their coping, which helps reduce their personal distress. Support for this tentative explanation comes from the insignificant association between personal and childrearing stress (i.e., items concerning the stress related to raising children) in the preterm mothers' group. This may suggest that some of the mothers in the premature infants' group, possibly the more reflective ones, were able to separate between the objective, day-to-day childrearing stress associated with the intensive care of the premature infant and their own subjective self-view of their maternal functioning, leading to less personal distress.

A different pattern appeared in the full-term group, where maternal self-focused PRF was linked with increased parents' personal stress. One interpretation of this finding may suggest that when raising a full-term infant, reflective mothers may experience and be cognizant of the personal distress associated with Stern's (72) concept of the “motherhood constellation.” Accordingly, motherhood involves grappling with acknowledging and adjusting to the major obligation involved in raising a child and the changes required in the mother's personal priorities, which may bring on some distress. Less reflective mothers of full-term infants may pay less attention to such mental processes. Instead, they may report less personal stress because of their tendency to focus on the day-to-day physical care of the infant, which may have become routine at this point. However, in the long run, poorer PRF may lead to less favorable outcomes for the mother and the child (35, 40).

Interestingly, and in line with previous research that showed differences in the way fathers' and mothers' PRF operates in the context of prematurity (59), we also found interesting gender differences in the premature group. Whereas, mothers' and fathers' PRF scores correlated positively among mothers and fathers of full-term infants, in the premature group they were unrelated. Particularly, mothers' and fathers' self-focused and child/relation-focused PRF were unrelated with each other in the preterm group. This finding suggests that fathers and mothers do experience and respond differently to prematurity. Furthermore, we found that PRF operated differently for fathers of premature infants compared to their spouses, and compared to fathers of full-term infants. Specifically, and as expected, fathers' PRF appeared to be a parental resource linked with less childrearing stress, but only in the context of raising a full-term infant. This finding is consistent with other studies also demonstrating that fathers' PRF is a protective factor for the parents (55) and for the child (54). In contrast, for the premature group, greater paternal PRF was linked with elevated childrearing stress. This finding echoes Ruiz's et al. (59) finding that reflective fathers of preterm infants tend to focus more on their personal needs and their childrearing responsibilities. Our findings suggest that this focus may increase their and their spouses' stress regarding childrearing obligations and priorities. It can be hoped that the combination of high reflection coupled with high stress may lead these fathers to seek help. Less reflective fathers of premature infants experienced less childrearing stress. However, as with mothers, their poorer PRF may lead these fathers to be more focused solely on daily routines (73) and less sensitive to the mothers' and the infants' mental needs. Such lack of sensitivity might jeopardize the infants' development (30, 74).

The finding of fathers' PRF as a significant moderator of the link between birth status and parenting stress is important given the scarce and inconsistent findings about the contribution of fathers' mentalization capacities and PRF to parenting and the child's outcomes (49, 52, 75). The moderating links of paternal PRF we found suggest that, at least in the case of fathers of full-term infants, the fathers' self-focused PRF served as a protective factor, as it was linked with less childrearing stress. A study using fMRI methods indicated that fathers who serve as primary caregivers for their children showed similarities in the activation of a global “parental caregiving” network, particularly the emotional processing and mentalizing networks. When interacting and observing video clips of their own parent-infant interactions, primary-caregiving fathers exhibited higher levels of amygdala activation (related to emotional processing), similar to those of primary care mothers, greater STS activation (related to mentalizing) and stronger functional connectivity between the amygdala and STS than less involved fathers. The primary care-giving fathers also demonstrated better cooperative and co-parenting skills (76). While we did not assess coparenting or the fathers' involvement, we may speculate that the fathers in our study who demonstrated strong mentalization skills, meaning high levels of PRF, at least those who parented a full-term infant, may have been more involved in caring for their infant. Their involvement may have helped reduce the parents' joint childrearing stress for them and for the mothers. Furthermore, highly reflective fathers may also interact in more emotionally meaningful ways with their infants than less reflective fathers. Given the transactional model that governs our thinking, the father's high level of PRF might increase his involvement with the child, which, in return can promote his infant's development of self-regulation skills, making parenting more satisfying (55).

Interestingly, the moderating effect of PRF for both the mothers and fathers was evident only for the self-focused PRF, not for the child/relation-focused PRF. This finding contrasts with Borelli's et al. (47) study, where mothers' child/relation-focused PRF moderated the link between the mother's arousal and her over-controlling behavior. This inconsistency may be related to the differences in the children's ages, the developmental stages of the parents and the specific outcomes assessed in these studies. Specifically, we focused on the early postpartum phase rather than older children and examined the parents' subjective parenting experiences, not their parental behavior. These factors may be more strongly linked with self-focused reflection. Finally, we found very strong connections between the two PRF factors for both the mothers and fathers. This finding suggests that at least at this early age, the two facets of PRF converge.

### Limitations of the Study and Future Directions

Our study contributes to the existing literature by providing evidence regarding the importance of the PRF of both the mothers and fathers for the parents' subjective experience of parenting, particularly parenting stress. However, several caveats need to be mentioned. First, our relatively small sample may have limited our ability to detect additional significant effects. Furthermore, our sample was homogeneous, consisting of heterosexual, highly educated cohabiting parents of premature and full-term infants. Thus, we must be cautious about generalizing the findings to other, more diverse, and more at-risk parents of premature infants, particularly parents of premature infants with congenital anomalies and other medical complications. Future studies can look at sicker infants as well as more diversified families including same-gender parents, single parents and parents who cope with significant economic hardships along with prematurity to broaden our understanding of the parenting experience with regard to prematurity. Another major limitation of our study was its cross-sectional design and the reliance on one-time assessment of PRF and parenting stress. Longitudinal, repeated-measures designs, following infants from birth throughout the first years of life, will provide a better understanding of causal and longitudinal effects of birth status on PRF and parenting stress. Such studies can also examine how birth status, PRF and parenting stress are linked over time and are associated with the socioemotional adaptation of parents of premature infants. Moreover, our study relied on subjective, self-report measures, which may be biased by social desirability as well as parents' conscious and unconscious denial strategies, particularly regarding parental stress. It is recommended that future studies include observational and direct measures of parental stress, as well as parents' mentalization skills. Research indicates gaps between parents' mentalization skills as expressed on interviews (as was done in our study) and their actual mentalizing behaviors with their infants (77). These may stem from the fact that the assessment of PRF depends on a parent's verbal skills and does not address its quality or utility in action. In some cases, high PRF may in fact reflect a tendency to hypermentalize, a non-adaptive tendency to focus exclusively on one's own internal state at the expense of external reality (30). Hence, future research can benefit from including observational measures of parental mentalization, such as the mind-mindedness (78) or the parental embodied mentalization assessment (77), as well as parent-child dyadic and triadic observations to test the associations between PRF and parenting behavior. Moreover, given the importance of fathers' involvement and the quality of coparenting and family atmosphere in the case of prematurity (10, 61), future studies may want to include measures of coparenting and fathers' involvement to examine the possibility that parents' PRF, particularly fathers', is linked with more emotional and childrearing involvement. Finally, we assessed parenting stress *via* a generic, self-report measure, which has been criticized as less sensitive in capturing actual stress and distress among parents of premature infants (26). Future studies can use questionnaires that address parenting stress in the context of prematurity more specifically and/or combine physiological and behavioral measures to assess parenting stress more directly.

### Clinical Implications

The understanding that at least some families experience intense stress and difficulties in dealing with prematurity, either by itself, or due to associated difficulties in the parents or in the child, has led to the devising of several important preventive and therapeutic early interventions. These methods have proven effective in reducing stress among parents of premature infants [e.g., (79–81)]. A meta-analysis which tested the effectiveness of interventions for parents of premature infants reported of reductions in the mothers' anxiety, depression, and parental sense of efficacy as well as some positive effects on the infants following these interventions. The authors argue that psychosocial support for the parent, parenting education and developmental support of the infant are the key components that account for the interventions' efficacy (82). Interestingly, the meta-analysis did not find significant changes in maternal stress following these interventions. One reason for the lack of impact on parental stress may be that often the interventions offered to parents of preterm infants are short-termed and some are limited to the hospitalization period (83). Our findings, which focused on parents 6–7 months postpartum and documented stress among some parents, particularly fathers, along with other studies that have identified interactive, relational and functional difficulties among premature infants and their parents, highlight the importance of providing preventive and therapeutic interventions and follow-ups to interested stressed parents of premature infants. Furthermore, mental health professionals who specialize in perinatal care and well-baby care providers can be trained to identify at-risk parents, particularly those who demonstrate low levels of PRF. Poor PRF often accompanies adult psychiatric psychopathology (45) and is linked with poor parenting and difficulty in creating benevolent parent-child relationships (30). Fortunately, there are several effective interventions for increasing PRF [see (74)] and parental mentalization skills in general. For example, the Minding the baby program (84) works to enhance PRF as well as improving affective communication between the mother and the child. The intervention provides intensive home visiting services, which may be particularly helpful for parents adjusting to a fragile premature infant. It may also provide the social support, which has been noted as central to effective interventions for parents of premature infants (82). Additionally, (85) recently published promising results of a parenting intervention which used a smartphone app and was specifically designed to increase mothers' attunement to their infant's mental states during the first 6 months of life. The intervention consisted of a brief, initial face-to-face meeting at baseline which provided the mothers with psychoeducational information about the importance of attuning to their infant's mental states. Following this session the intervention mothers received daily prompts with developmental information and an invitation to reflect on their infant's mental states by sending in photographs and video-clips which indicate their attention to their infant's internal states. A research team provided feedback to the mothers which confirmed, expanded and encouraged further minding of the infants' mind on the part of the mother. Following the 6 months of the intervention, the mothers who used the app (the intervention group) demonstrated more frequent and more accurate mental state comments when interacting with their infants, i.e., better mentalization skills, compared to a control group who did not use the app. Delivering an intervention *via* a smartphone app which supports and enhances parental mentalization skills may be especially useful and handy for parents of a premature infant who may find it difficult to travel to a clinic or an infant developmental center. Finally, it has been noted that many of the existing interventions target mothers only (82, 86) and are less committed to involving resistant fathers in the therapy. Our findings, along with other studies, indicate the important contribution of fathers to the family's and the couple's parenting and emotional experiences. Thus, it is important to include fathers in the intervention and make the needed arrangements to engage them in the process. Moreover, our findings show that a father's PRF works differently than a mother's PRF. Thus, we recommend that therapists who work with couples insist on both parents' participation. In addition, therapists need to adopt a mindful and flexible therapeutic stance in order to observe, infer and adapt the work to meet the mentalization styles and needs of the two parents.

## Data Availability Statement

The raw data supporting the conclusions of this article will be made available by the authors, without undue reservation.

## Ethics Statement

The studies involving human participants were reviewed and approved by the Tel Aviv Sourasky Medical Center IRB approval TLV-0287-13 and Tel Aviv-Yaffo Academic College IRF 2014024 approval. The patients/participants provided their written informed consent to participate in this study.

## Author Contributions

All authors contributed to conception and design of the study, data collection, data analysis, contributed to manuscript writing and manuscript revision, read, and approved the submitted version.

## Funding

This study was supported by an internal grant from the Academic College of Tel Aviv-Yaffo.

## Conflict of Interest

The authors declare that the research was conducted in the absence of any commercial or financial relationships that could be construed as a potential conflict of interest.

## Publisher's Note

All claims expressed in this article are solely those of the authors and do not necessarily represent those of their affiliated organizations, or those of the publisher, the editors and the reviewers. Any product that may be evaluated in this article, or claim that may be made by its manufacturer, is not guaranteed or endorsed by the publisher.
